# Experimental Investigation on Ductile Mode Micro-Milling of ZrO_2_ Ceramics with Diamond-Coated End Mills

**DOI:** 10.3390/mi9030127

**Published:** 2018-03-14

**Authors:** Rong Bian, Eleonora Ferraris, Yinfei Ynag, Jun Qian

**Affiliations:** 1 Industrial Center, Nanjing Institute of Technology; Nanjing 211167 China; 2 Jiangsu Key Laboratory of Precision and Micro-Manufacturing Technology, Nanjing University of Aeronautics and Astronautics; Nanjing 210016, China; yangyf@nuaa.edu.cn; 3 Department of Mechanical Engineering, KU Leuven & Member Flanders Make, Leuven 3001, Belgium; Eleonora.Ferraris@kuleuven.be (E.F.); jun.qian@kuleuven.be (J.Q.)

**Keywords:** micro-milling, ductile, diamond-coated, zirconia, cutting force, tool wear

## Abstract

ZrO_2_ ceramics are currently used in a broad range of industrial applications. However, the machining of post-sintered ZrO_2_ ceramic is a difficult task, due to its high hardness and brittleness. In this study, micro-milling of ZrO_2_ with two kinds of diamond-coated end mills has been conducted on a Kern MMP 2522 micro-milling center (Kern Microtechnik GmbH, Eschenlohe, Germany). To achieve a ductile mode machining of ZrO_2_, the feed per tooth and depth of cut was set in the range of a few micrometers. Cutting force and machined surface roughness have been measured by a Kistler MiniDynamometer (Kistler Group, Winterthur, Switzerland) and a Talysurf 120 L profilometer (Taylor Hobson Ltd., Leicester, UK), respectively. Machined surface topography and tool wear have been examined under SEM. Experiment results show that the material can be removed in ductile mode, and mirror quality surface with Ra low as 0.02 μm can be achieved. Curled and smooth chips have been collected and observed. The axial cutting force *F_z_* is always bigger than *F_x_* and *F_y_*, and presents a rising trend with increasing of milling length. Tool wear includes delamination of diamond coating and wear of tungsten carbide substrate. Without the protection of diamond coating, the tungsten carbide substrate was worn out quickly, resulting a change of tool tip geometry.

## 1. Introduction

### 1.1. Background

Thanks to the favourable combination of outstanding mechanical, thermal, and chemical properties, technical ceramics, such as oxides, carbides, nitrides, and borides, have received increasing attention in the recent years, and find broad applications in the modern industry [1]. Specifically, zirconium oxide (ZrO_2_) is the second most-used ceramic on the market; it provides remarkable fracture toughness, exceptionally good thermal insulating properties and ionic conductivity, and it is also biocompatible [2]. Main applications of this material include pump impellers for turbo-machinery, diesel injection micro nozzles, micro-fluidic devices, micro-moulds, oxygen sensors for foundry industry, dental and orthopaedic implants, etc.

However, manufacturing components of ceramic at a hardened state, in general, is a difficult task. Several research works have been done in the past. Some traditional abrasive based surface finishing processes, such as grinding and lapping, are commonly applied. But they are reported to cause flatness-related error and subsurface damage on machined surface [3]. To achieve damage-free surface finishing, ductile-mode machining has been presented as a viable alternative to traditional finishing processes for brittle materials such as glass, silicon, and tungsten carbide in the past few years [4,5,6,7,8,9]. For ceramics, most applied machining methods are diamond turning and grinding. Bifano et al. [10] performed ductile regime grinding process on several ceramics, and found that critical un-deformed chip thickness is a function of intrinsic material properties governing plastic deformation and fracture. Yan et al. [11] investigated the feasible machining of silicon carbide ceramics by single point diamond turning with large nose radius (10 mm). Beltrão et al. [12] achieved ductile mode machining of different kinds of commercial PZT ceramic samples. Zhong [13] reported ductile or partial ductile mode grinding of some brittle materials, including ZrO_2_, and found ductile streaks on the machined surface. Furthermore, Zhao [6] and Yan [5] discussed ultrasonic assisted ductile grinding of nano-engineered ZrO_2_ ceramics, and achieved the theoretical critical depth. Though high-quality surface finishing on ceramics can be achieved by ductile mode grinding and turning, they are really time consuming, and still limited in machining some complex three-dimensional features for micro-moulds.

Some other machining methods also have been applied to achieve better machining performance. M. Kumar et al. [14] studied laser-assisted micro grinding process for a hard silicon nitride ceramic, to evaluate the feasibility of using the thermal cracking mechanism to micro machine hard ceramics. Toru Kizaki et al. [15] also conducted laser-assisted machining of fully sintered zirconia ceramics using a diamond bur. They were considered to have higher material removal rates and lower grinding force and tool wear. Ferraris et al. deeply investigated the machining behaviour of many electrically conductive ceramics, including Al_2_O_3_-, ZrO_2_- and Si_3_Ni_4_-based ceramic composites via electrical discharge machining (EDM) [16,17]. Although complex shapes can be machined by micro EDM, it is not applicable to many other non-conductive ceramics. Furthermore, the machined surface with thermal cracks may not meet the request of precision components. 

### 1.2. Ductile Mode Micro-Milling

As a direct scale-down of normal milling process, micro-milling brings in new flexibility in micromachining, due to its capability of creating three-dimensional small features with relatively high material removal rate [18,19,20]. It has already been used in many applications, such as micro parts in watches, guiding components for minimal invasive surgery, housing of micro engines, parts for micro injection moulding, and housing and packaging solutions for micro optics and micro fluidics devices [21]. Despite the many advantages of micro-milling, there are still challenges to overcome, especially in the case of dealing with brittle and hard materials, such as ceramics. Firstly, the cutting force can be relatively higher, so stiffer miniature/micro mills are required in order to prevent tool breakage and deflection, which have a negative influence on the cutting process [22,23]. Furthermore, the hardness and abrasive resistance of traditional tungsten carbide tool materials is insufficient for machining hardened ceramics. The tool shape deteriorates quickly, due to the wear of the cutting edges. Therefore, it is necessary to apply on the tool ultra-hard materials, such as cubic boron nitride (CBN), poly crystalline diamond (PCD), and diamond coating, which exhibit superior mechanical and tribological properties [22,24,25].

Successful attempts on implementing ductile milling on hard and brittle materials, such as engineering ceramics and carbide, have been conducted recently [26,27,28]. Matsumura and Ono [29] reported that grooves with axial depth of cut in the range of 15–20 μm can be machined in glass if the CBN ball end mill is tilted at a certain angle in the feed direction. Bian et al. [30] investigated the feasibility of meso-scale hard milling of ceramic with Ø2 mm diamond-coated end mills, and presented a mirror-like machined surface and a 3-dimensional structure. Cheng et al. [31] even achieved nanometric surface finish and micro-rib with a width–depth ratio of 1:10 on tungsten carbide by ductile mode milling with micro PCD tools. Thus, ductile mode micro-milling seems a feasible way to achieve complex shapes and crack-free surfaces for brittle and hard materials. However, there is still a noticeable lack of experience in this specific topic, especially in micro-milling of ceramics in hard state.

In this paper, an experimental study of micro-milling of fully sintered ZrO_2_ ceramics with diamond-coated end mills has been carried out. The primary objective is to provide suitable machining parameters for micro-milling of ZrO_2_ ceramics, and elaborate the applications of diamond-coated tools and eventually evaluate the process characteristics in ductile milling of ZrO_2_ ceramic, for instance, surface roughness, tool wear, cutting forces, and material removal mechanisms.

## 2. Experimental Set-Up

### 2.1. Work Piece Material

The workpiece material used in this study is a commercial ZrO_2_ ceramic, mainly consisting of insulating tetragonal polycrystalline zirconium oxide (Y-TZP) partially stabilized with yttria. The material is ready after a final sintering process at a temperature of 1350–1500 °C, and provides a high hardness of about 1200 HV10 and a relatively high fracture toughness. Table 1 lists the chemical composition and mechanical properties of the material.

### 2.2. Machine Tool and Micro End Mills

The machining tests have been conducted on a Kern MMP 2522 micro-milling centre. This equipment is specifically designed for micro-milling applications; it is constructed with a C-frame and a polymer-based dumped basement for higher accuracy and stability; the machine is capable of achieving positional and repetition accuracy of ±1 μm, and it is equipped with an infrared touching probe for part alignment, and a BLUM laser scanning device (Blum-Novotest GmbH, Grünkraut, Germany) for tool measurement and geometrical compensation. The tool spindle can rotate up to 40,000 rpm.

**Figure 1:**
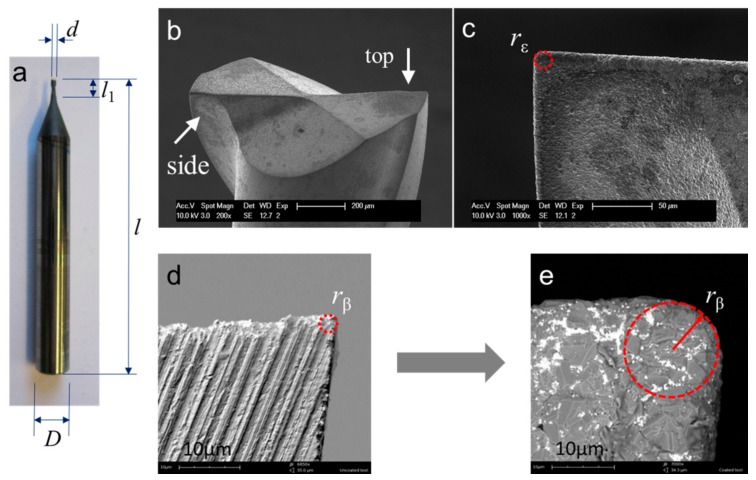
SEM images of the micro end mill: (**a**) overview of the geometry of the end mill; (**b**) top view of the cutting-edge radius; (**c**) side view of the tool tip; (**d**) the original cutting-edge radius before diamond coating; (**e**) the cutting-edge radius after diamond coating.

**Figure 2:**
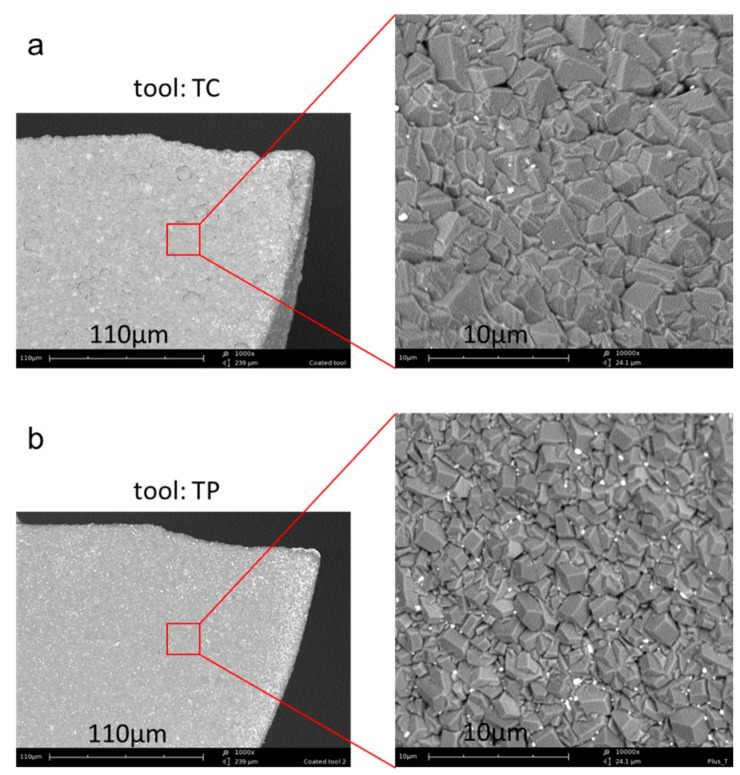
Surface morphology of the two kinds of diamond coating: (**a**) TC—conventional diamond coating; (**b**) TP—nanocrystalline diamond coating.

The cutting tools used are 2-flute flat-bottom micro end mills with nominal diameters of Ø1 mm, from VAN Hoorn (Van Hoorn Carbide, Weert, The Netherlands). Figure 1a shows the main tool geometry. The tools have a big tool shank diameter of Ø6 mm, and a short tool neck of 4 mm to improve their stiffness. The helix angle is about 30°. The designed rake angle and relief angle is about 2° and 14° respectively (listed in Table 2). These tools are made of fine grain tungsten carbide (grain size ~0.5 μm), and are further coated by Oerlikon Balzers (OC Oerlikon group, Balzers, Liechtenstein) with chemical vapor deposition (CVD) diamond of 6–7 μm in thickness. Two different kinds of CVD diamond coating were used. As shown in Figure 2, the first one marked with TC features a normal crystalline coating structure, and protects the tools against abrasive wear, while the second type marked with TP features a relatively smooth surface with nanocrystalline coating structure, and is expected to show better adhesive wear resistance. They had the similar micro hardness and service temperature. The average surface roughness of the two kinds of diamond coating were measured to be about Sa 237 nm and Sa 182 nm, respectively, by a white-light-interferometer profiling system, Wyko NT3300 (Veeco Instruments Inc., Plainview, NY, USA).

All new micro-tools have been first inspected with SEM to check the integrity of the diamond coating. An overview of the tool geometry is shown in Figure 1b–e. The tool tip corner radius *r*_ε_ is estimated to be about 8 ± 0.5 μm in the SEM picture from the side view (Figure 1c). Figure 1d,e shows the cutting-edge radius *r*_β_ before and after the tool was coated. Obviously, the diamond coating increased the cutting-edge radius *r*_β_ from a very small value, about 1~2 μm, to a bigger value, about 7.5 ± 0.5 μm, which is normally larger than the selected uncut chip thickness in ductile milling of ZrO_2_, so the cutting edge always works with a negative rake angle during the cutting process.

### 2.3. Experimental Conditions and Procedures

**Figure 3:**
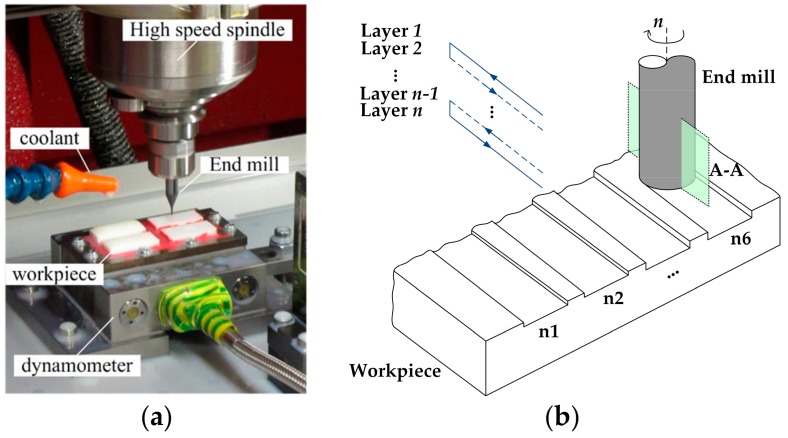
View of (**a**) experimental set-up and (**b**) groove milling sketch.

An experimental set-up has been prepared as shown in Figure 3a. Fully sintered ZrO_2_ workpieces were attached on the fixture with wax, and the surfaces of the samples were precisely ground to insure flatness and alignment. The fixture was then mounted on a dynamometer (Kistler Minidyn9256C1, Kistler Group, Winterthur, Switzerland) used to measure the cutting force. Full-width milling of 11 mm long straight grooves has been carried out with various levels of cutting parameters (Figure 3b). The spindle rotating speed n was fixed at 38,000 rpm, which corresponds to a constant cutting speed *v*_c_ about 120 m/min. Three levels of feed per tooth, *f*_z_, (1, 3, and 5 μm per flute) and two levels of depth of cut, *a*_p_, (2 and 4 μm) were chosen to evaluate the effect of cutting parameters on machining process. The full experiment matrix is listed in Table 3. During each test, a newly examined end mill was used, and six groove samples marked from n1 to n6 after different cutting length were kept for inspection. As listed in Table 4, groove n1 and n2 after short cutting length were considered to be machined under new tool condition. Groove n3 to n6 machined at relatively longer cutting length were used to estimate the effect of tool wear on the machined surface quality.

**Figure 4:**
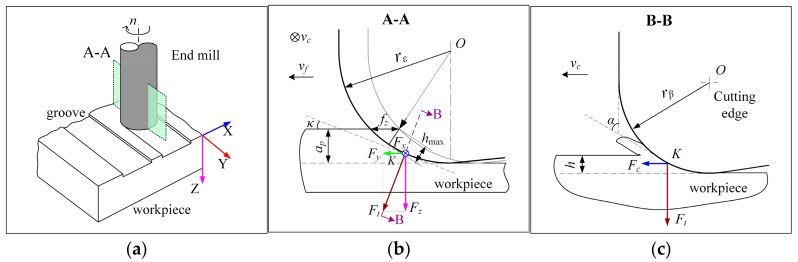
Schematic geometry in cutting zone, (**a**) sketch map of groove milling; (**b**) schematic geometry from A–A cross-section; (**c**) schematic geometry from B–B cross-section.

Due to the cutting parameters used in the experiments being very small, micro end milling shows different cutting geometry from conventional milling. Figure 4 shows the specific geometry of cutting zone. As shown in the cross-section A–A (Figure 4b), due to the axial milling depth of cut *a*_p_ is smaller than the tool corner radius *r*_ε_; actually, only a small part of the cutting edge that located on the bottom of tool corner works for cutting. For the specific selected *f*_z_ and *a*_p_, the maximum uncut chip thickness, *h*_max_, can be approximately calculated by the Equation (1), when $\sqrt{2Ra_{p}-a_{p}{}^{2}}>f_{z}$ [8].

(1) $$h_{\max}=r_{\varepsilon }-\sqrt{r_{\varepsilon }{}^{2}+f_{z}{}^{2}-2f_{z}\sqrt{2r_{\varepsilon }a_{p}-a_{p}{}^{2}}}$$

In this study, the calculated maximum uncut chip thickness, *h*_max_, (listed in Table 3) is much smaller than the tool cutting-edge radius, *r*_β_ (7.5 ± 0.5 μm), so the tool cutting edge actually works with a big negative rake angle, *γ* (Figure 4c), which are traditionally reported to be beneficial for machining hard materials. They promote the formation of large compressive stress into the chip formation zone, and reduce the trailing tensile stresses, thus obstructing crack propagation of pre-existing induced flaws and defects, while enhancing the plastic deformation of the undergoing material, and therefore, a ductile chip formation [8,32].

All the milling tests were conducted in wet conditions using a water-based emulsion to remove chips and debris. After milling tests, the surface roughness, Ra, of the bottom of the milled grooves has been measured on a Talysurf 120 L profilometer along the centre line. The tool wear in the final milling state and machined surface quality were inspected by a SEM Philips XL40 FEG (Philips, Eindhoven, The Netherlands).

## 3. Results and Discussion

### 3.1. Surface Roughness

**Figure 5:**
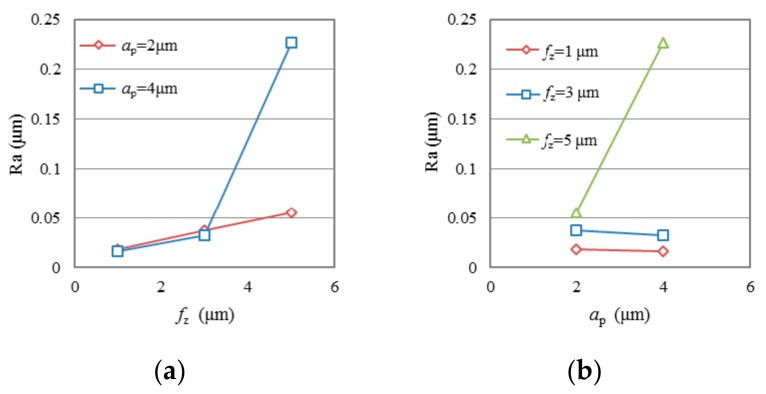
Surface roughness Ra as a function of (**a**) *f*_z_ and (**b**) *a*_p_ in test group TC.

**Figure 6:**
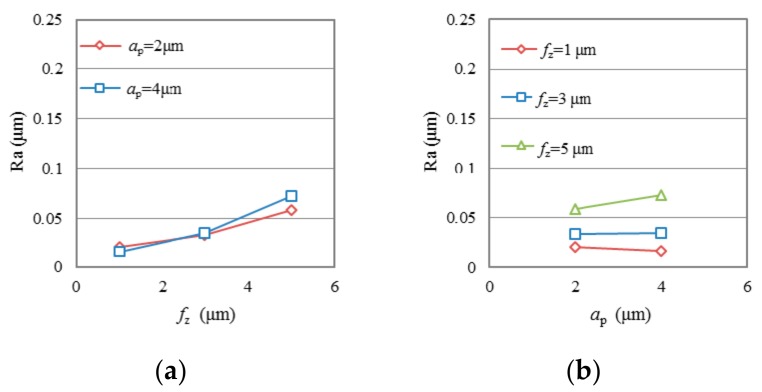
Surface roughness Ra as a function of (**a**) *f*_z_ and (**b**) *a*_p_ in test group TP.

To reduce the effect of tool wear, only the first two groove samples (n1 and n2) machined after very short cutting length (*l*_ac_ < 132 mm) in each test were selected to evaluate the effect of cutting parameters on surface roughness. Figure 5 and Figure 6 show the variation of average surface roughness Ra (average value of groove n1 and n2 in each test) as a function of *f*_z_ and *a*_p_ in test group TC and group TP, respectively. It is seen that the use of both conventional diamond coating (TC) and nano diamond coating (TP) gives good surface finish. The surface roughness, Ra, generally presents an increasing trend with the increase of *f*_z_. Average Ra ranges of 0.018 μm to 0.0376 μm can be achieved when *f*_z_ is at low level. Only in test TC2, when both the *f*_z_ and *a*_p_ are at high level, did Ra increase dramatically to a very high value, about 0.227 μm. The test results do not show any clear law on the effect of milling depth *a*_p_ on Ra. As shown in Figure 5b and Figure 6b, when *f*_z_ is at low level, Ra does not change, or slightly decreases with the increasing of *a*_p_; when *f*_z_ is high, Ra increases with the increasing of *a*_p_.

**Figure 7:**
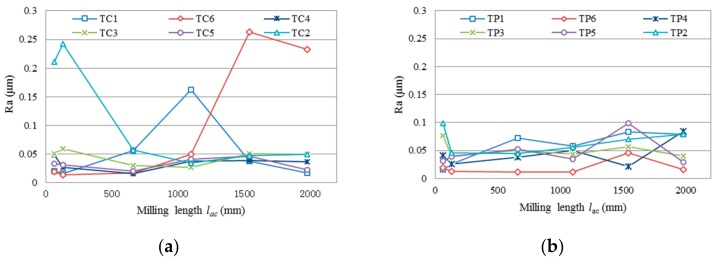
Surface roughness Ra verses milling length, (**a**) in test group TC; (**b**) in test group TP.

Figure 7 shows the average surface roughness Ra as a function of milling length in each test. Specifically, most of the Ra values in test group TC are within the range between 0.014 μm and 0.059 μm, with an exception of a few above 0.15 μm. All Ra values in test group TP are range between 0.012 μm and 0.099 μm. It is observed that the Ra of the machined surface both in test group TC and TP changes randomly with the increase of milling length. The reason for this irregularity may be the randomness of tool wear of the diamond-coated end mills.

### 3.2. Tool Wear

**Figure 8:**
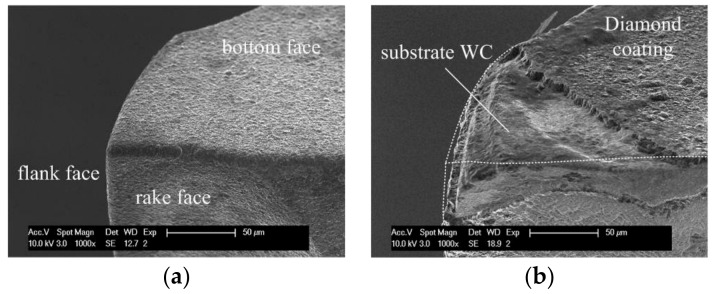
Comparison of (**a**) a new tool edge and (**b**) a typical worn out tool edge.

In the machining processes, various wear conditions, such as trivial coating delamination on the tool tip, big delamination of coating, and even wear of tool substrate, WC, have been observed. Figure 8 shows a comparison between a new tool tip and a typical worn tool tip of the conventional diamond coated end mill. As shown in Figure 8b, the delamination of diamond coating took place on the rake face, flank face, and bottom face. Obvious attrition wear on the exposed substrate WC can be also observed. The tool wear changes the specific tool tip geometry, results in a blunt cutting edge and a bigger effective tool corner radius.

**Figure 9:**
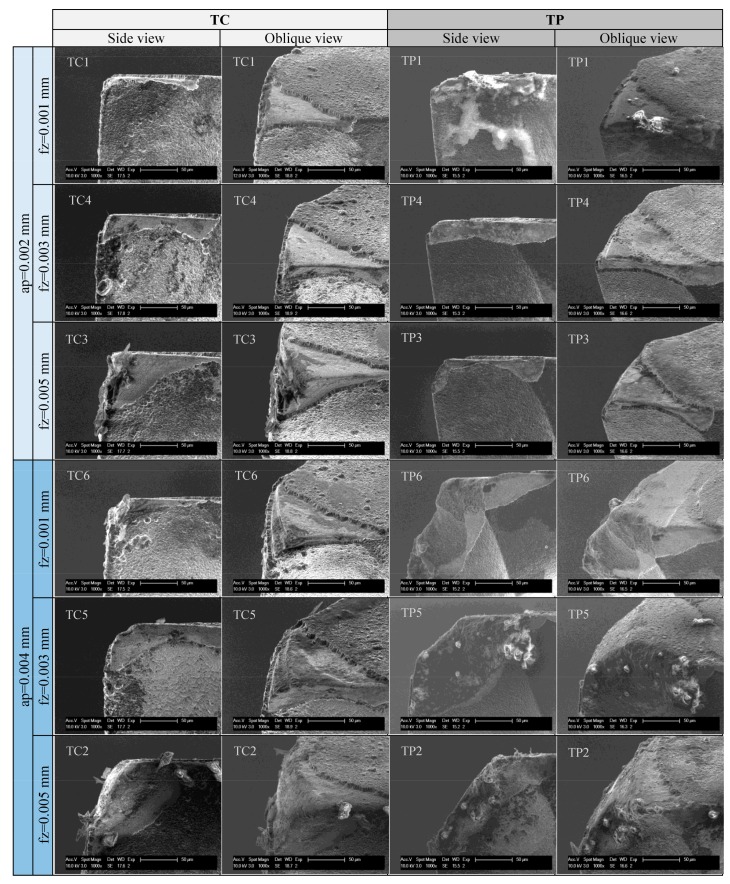
Tool tip wear of the conventional diamond-coated tool—TC and nano diamond-coated tool—TP.

Figure 9 presents the worn tool tips after the same cutting length of 1.98 m under different cutting parameters in both test group TC and TP. From the side view in Figure 9, it is found that the delamination area of diamond coating in rake face is considerably affected by the cutting parameters. When the feed per tooth *f*_z_ changed from 1 µm to 5 µm, the diamond coating delamination area on the rake face presented an increasing trend. Consequently, the WC substrate had the similar trend of wear, due to the lost protection of diamond coating. Especially under the high level *a*_p_ of 0.004 mm, severe attrition wear of exposed WC in test group TC can be seen. In test group TP, more serious tool wear phenomenon can be found on the body of nano diamond-coated end mills. In the exposed WC substrate fractured directly after the diamond coating delamination, clear fracture surface morphology can be seen on the tool tip. These wears or fractures produce a blunt tool corner and a reduction of effective milling diameter, which will affect the machining quality and accuracy of the grooves. It can be inferred that the nano diamond coating in this study is less robust than the conventional diamond coating, and may fall off faster during the milling process. Thus, the substrate WC loses its protection prematurely, and therefore, wears and breaks more seriously.

It is also observed from the pictures that there were still some microchips together with the coolant adhered on the surface of tool tip, although the tools had been already cleaned in an ultrasonic bath for 20 min. This may also cause negative effects on the milling process. It is suggested that better chip removal methods should be employed.

### 3.3. Cutting Force

**Figure 10:**
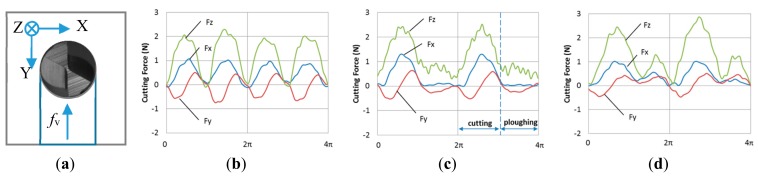
Typical milling force signals in the test, (**a**) coordinate system description; (**b**) evenly milling by two cutting edges; (**c**) single edge cutting; (**d**) unevenly milling by two cutting edges.

In the study, milling force has been measured by a dynamometer to help understand the real engagements between cutting tool and workpiece. The acquired data was filtered with a low pass filter with a cut-off of 2 KHz. This is expected to leave the force signal unaffected with regard to the harmonics generated by the cutting process. A normal cutting force in time domain is shown in Figure 10b. In one rotation, there should be two engagements between the cutting edges and the workpiece. For each engagement, the force variation is a result of the change of the chip load during the tool rotation. The tool angle of 0 is defined as the instantaneous point that the tool tip comes into contact with the workpiece. At that point, the force in *X* direction, *F*_x_, and in *Y* direction, *F*_y_, will be zero. Then, the *F*_x_ increases to a maximum value, and then goes down to zero again, while the *F*_y_ firstly goes to a negative maximum, then increases to a positive maximum, and finally goes back to zero. The cutting force in *Z* direction, *F*_z_, has a similar behaviour to *F*_x_.

However, it is seen that during the initial stage of some tests, there is only one pick force in one rotation (Figure 10c), which indicates that only one tool edge cuts materials away in one rotation, and there is only a ploughing process instead of cutting between the other tool edge and the workpiece. Sometimes it is also seen that a big pick force is followed by a small pick force (Figure 10d), which indicates that one of the cutting edges removed more materials than the other one. The reason behind this is that the feed per tooth, *f*_z_, used in ductile micro-milling is very small, which is close to the diameter run-out of the spindle. The cutting edge with a bigger effective rotating radius will cut more material than the other one. Thus, single tool edge-cutting or uneven cutting phenomenon is observed.

**Figure 11:**
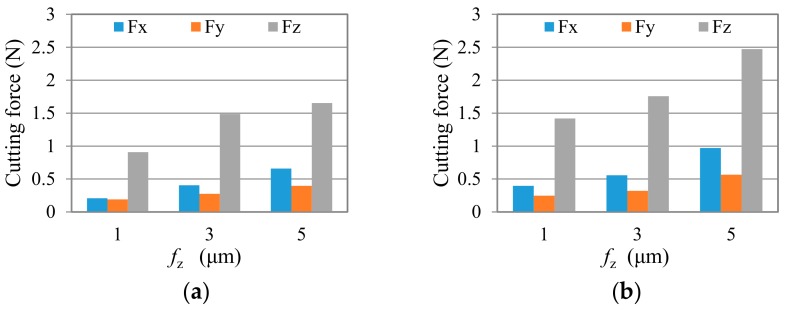
Cutting force in the test group TC after cutting length of less than 55 mm, (**a**) *a*_p_ = 2 μm, and (**b**) *a*_p_ = 4 μm.

**Figure 12:**
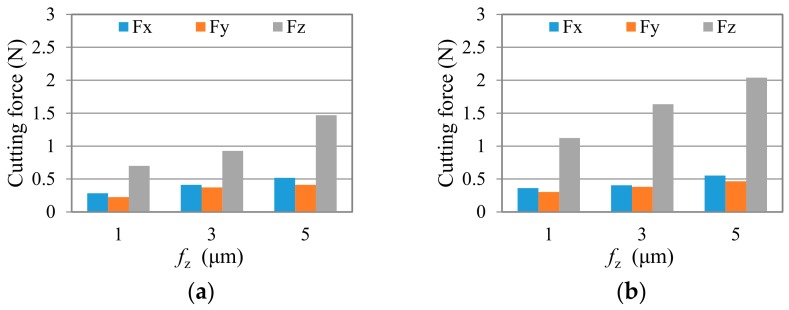
Cutting force in the test group TP after cutting length of less than 55 mm; (**a**) *a*_p_ = 2 μm, and (**b**) *a*_p_ = 4 μm.

In this investigation, the maximum of absolute force peak values was chosen to evaluate the cutting load on cutting direction and feed direction. Figure 11 and Figure 12 show the average peak cutting forces in each test after a short cutting length of less than 55 mm. Generally speaking, the cutting force is very small (~0.2 N to ~2.47 N), due to the tiny removal of material in each cutting process. It can be seen that the cutting forces in all three directions, especially in Z direction, present a trend of going up with the increase of the feed per tooth, *f*_z_, and axial milling depth, *a*_p_. For all of the tests, *F*_z_ is much larger than *F*_x_ and *F*_y_. This is because that the depth of cut, *a_p_,* used in the study is smaller than the tool corner radius, *r_ε_*, which makes the cutting edge work with a small major cutting angle, *κ* (see Figure 4c). Thus, a bigger axial component is formed. As illustrated in Figure 4, when the cutting edge is moved to the top of the groove (immersion angle of about 90 degrees), the milling force at the point *K* is as shown in Figure 4b. At this instant, the cutting force F_c_ is mainly related to *F*_x_, and thrust force F_t_ is synthesized by *F*_y_ and *F*_z_. Due to the fact that *F*_z_ is much bigger than *F*_x_ and *F*_y_ in this test, it can be drawn that the bigger *F*_z_ here indicates that the thrust force is much larger than the cutting force, as shown in the B–B cross-section view in Figure 4c. This result agrees with the research of Liu [7]. Therefore, large compressive stress is generated in the chip formation zone, whereby the crack propagation is suppressed, and dislocation motion becomes the predominant activity, which leads to material removal by plastic deformation during ductile-mode machining [33]. Comparing the results of cutting force in two test groups, it is seen that the cutting force in group TP is a little smaller than that in group TC when under the same cutting parameters. This is mainly due to the smoother surface of the nano diamond coating used in group TP, which produces less force in the interacting process with the workpiece.

**Figure 13:**
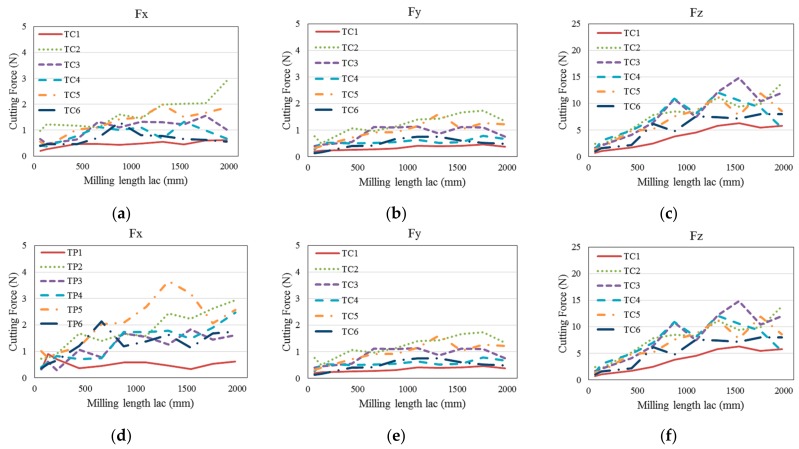
Cutting force in three directions changes with milling length: (**a**) TC—*F*_x_; (**b**) TC—*F*_y_; (**c**) TC—*F*_z_; (**d**) TP—*F*_x_; (**e**) TP—*F*_y_; (**f**) TP—*F*_z_.

Figure 13 shows the cutting force in three directions changes with the increasing of milling length in test groups TC and TP. It is seen that with the increase of milling length, the cutting forces, especially in *Z* direction, present a rising trend with some random fluctuations. For example, the *F*_z_ changes from a minimum 1.6 N to about 12 N in test TC3, and from 1.4 N to about 23 N in test TP6. This phenomenon can be attributed to the uncertainty of the tool wear in milling process. Accidental delamination of diamond coating, fracture of WC substrate, and brittle failure of the machining surface, may all lead to changes in cutting force. The wear on the bottom surface of the cutting tools increases the contact area between the tool and the workpiece, which also leads to a dramatic increase in *F*_z_.

It also can be seen that unlike in the initial milling stage, with the increase of the milling length, the cutting forces in test group TP become larger than that in test group TC at the later milling stage. It can be inferred from Figure 9 and Figure 13 that the premature delamination of the nano diamond-coated tool leading to premature exposure of the substrate WC, results in more severe tool wear in test group TP than that in test group TC.

### 3.4. Material Removal Mechanism

**Figure 14:**
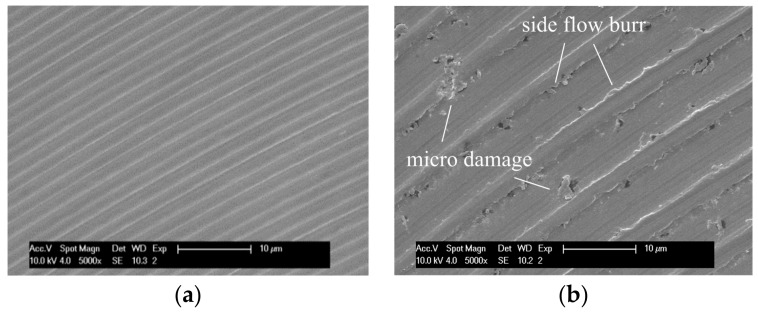
Machined surfaces of groove n1 in test (**a**) TC1: *f*_z_ = 1 μm, *a*_p_ = 2 μm; (**b**) TC2: *f*_z_ = 5 μm, *a*_p_ = 4 μm.

When machining hard and brittle materials, it is generally accepted that ductile behaviour is achieved when the specific material removal unit is small enough. The critical depth of cut *d*_c_ is specified as in Equation (2):

(2) $$d_{c}=0.15\left(\frac{E}{H}\right){\left(\frac{K_{\mathrm{IC}}}{H}\right)}^{2}$$

where *K*_IC_ is the fracture toughness, *H* is the hardness, and *E* is the elastic modulus [10]. For the ZrO_2_ used in this study, the critical depth of cut *dc* is calculated to be about 1.9 μm. The calculated maximum uncut chip thickness *h_max_* in the test ranges from 0.62 μm to 3.55 μm (see Table 3). According to the above principle, in tests TC1, TC4, TC6, TP1, TP4, and TP6, where the calculated *h_max_* is smaller than the critical *d_c_*, the machining should be in ductile mode, while in tests TC2, TC3 TC5, TP2, TP3, and TP5, brittle damages may take place because of the oversized uncut chip thickness. However, almost all the machined surfaces in the initial stage show ductile material removal mechanism, except in test TC2. Figure 14 compares the machined surface of groove n1 in test TC1 with *h*_max_ of 0.62 μm, and TC2 with *h*_max_ of 3.55 μm. It is seen that the machined surface in TC1 (Figure 14a) exhibits regular and smooth feed marks without any surface defect. However, in Figure 14b, though the surface texture shows plastic deformation, there are some obvious micro surface damages. Some micro side-flow burrs also can be observed. Under the cutting loads, the material which fails to form a chip is subjected to sufficiently high pressure to cause the material to flow to the side [34]. The side-flow burr will cause considerable increasing of the surface roughness, which may be the reason of the uncommon Ra value in test TC2.

It is also noticeable that in both images, the main feed marks matched well with the feed per rotation of the spindle instead of feed per tooth. This corresponds to the analysis before that only one cutting edge is actually working, or one cutting edge removed more material than the other one per rotation of the end mill.

**Figure 15:**
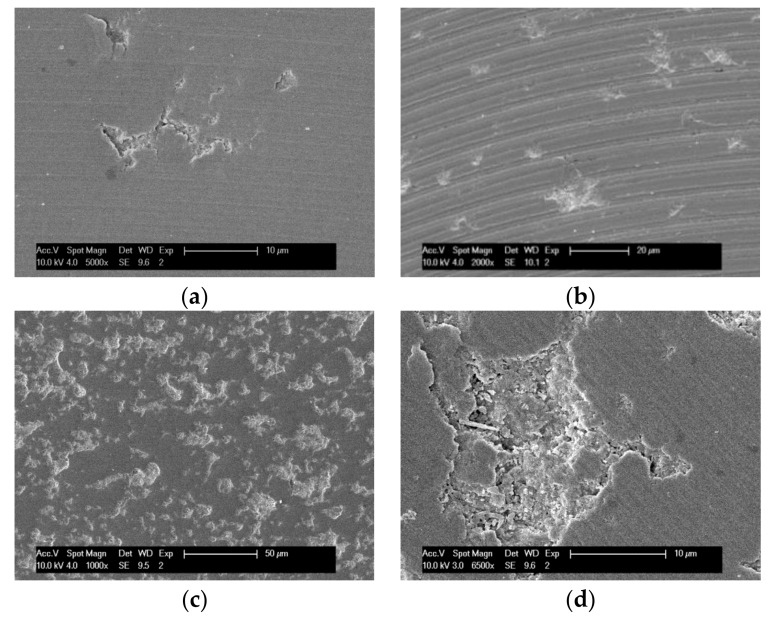
Typical machined surfaces with brittle damage: (**a**) tiny flaws; (**b**) scattered micro-pits; (**c**) large areas of brittle failure; (**d**) specific damage area.

With the increasing of cutting length, the machined surfaces show evidently different material removal mechanisms in some tests, due to tool wear. Figure 15 shows SEM images of some typical machined surfaces. For example, some small surface flaws (Figure 15a), sporadic scattered micro-pits (Figure 15b), and the distribution of large areas of brittle failure (Figure 15c) can be seen on the machined groove surface. A specific brittle damage area is shown in Figure 15d, the micro cracks and fractured features significantly affected the surface quality. These typical machined surface defects occur in different milling time. The main reason behind this is that tool wear aggravates with the increasing of cutting length. Without the protection of diamond coating, the tungsten substrate wears fast, and results in a change of tool tip geometry to form a blunt cutting edge which fails to remove material in ductile mode.

**Figure 16:**
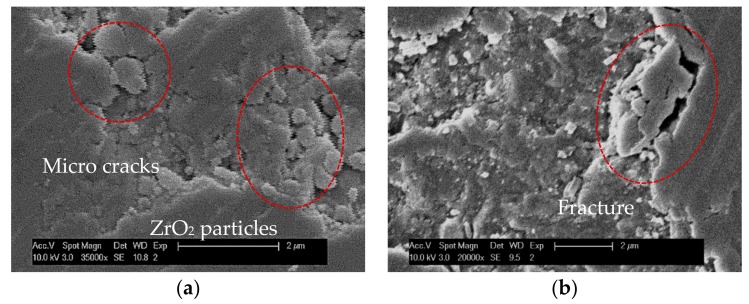
Machined surface flaws: (**a**) crushed in to particles; (**b**) fractured from edge.

Usually, the tiny brittle damage in machining process is difficult to detect in time. The surface with brittle failure will continue to be machined by the end mill. When the cutting edge meets the surface with brittle flaws, the tool edge will squeeze the damaged area, so that the zirconia ceramic may be crushed into particles, or may be fractured away from the edge of the pit, due to the extension of cracks, as shown in Figure 16.

### 3.5. Chips Shape

**Figure 17:**
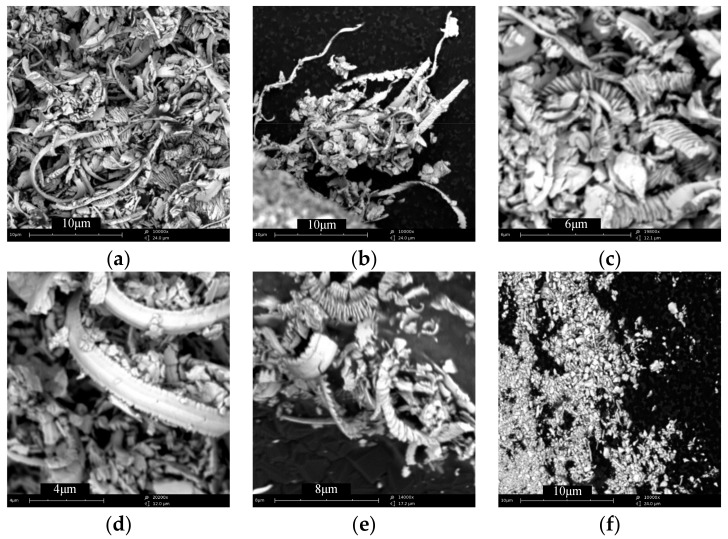
SEM images of chips: (**a**,**b**) long and continuous chips; (**c**–**e**) ductile chip with smooth back surface; (**f**) short and fractured chips.

The chips produced in the processes have been collected and then examined by SEM, as shown in Figure 17. The long chips shown in Figure 17a,b are collected at the initial stage of the milling process. It can be seen that the chip was probably formed in a same way as those in cutting ductile materials, where materials were removed from the workpiece by dislocations that generate the chip in layers contacted to each other. It can be seen from Figure 17c–e that the continuous chip is curled, and the chip back surface slides out from the rake face of the cutting tool and plastically deforms under the action of extrusion and friction to form a relatively smooth surface. The small and short powders in Figure 17f were collected at the later milling process, and might be formed by crack propagations in fracture processes.

## 4. Conclusions

This paper presents an experimental investigation on micro-milling of ZrO_2_ ceramics with diamond-coated end mills, delivering experimental evidence on performance of diamond-coated micro end mills in ceramic machining, related tool wear mechanisms, achievable surface roughness, cutting forces, material removal mechanisms, and so on. The following specific conclusions can be drawn from this investigation:

- The ZrO_2_ ceramics can be directly machined by diamond-coated end mills in ductile mode. Mirror quality surface around 20 nm Ra can be achieved when suitable parameters are selected. With the increasing of cutting length, the machined surface roughness changes irregularly due to tool wear.
- The tool wear includes diamond coating delamination and wear of substrate tungsten carbide. The coating delamination area is affected to a large extent by feed rate. The nano diamond coating in this study is less robust than the conventional diamond coating, and falls off faster. Without the protection of diamond coating, the tungsten substrate wears fast, and will change the tool tip geometry, blunting the tool cutting edge. The premature delamination of diamond coating is a limitation for its application in micro-milling of ceramics.
- Due to the small feed per tooth selected in micro-milling of ZrO_2_ close to the run-out of spindle, single tool edge cutting or uneven cutting phenomenon is observed. The cutting force *F*_z_ is normally bigger than *F*_x_ and *F*_y_, because of the small effective major cutting-edge angle. This results in considerably high compressive stress in the cutting zone. With the increase of cutting length, the cutting force presents an increasing trend with random fluctuations, due to the tool wear.
- When the un-cut chip thickness is small enough, smaller or even a little bigger than the critical *d*_c_ in experience, the ductile machined surface is still available. Chips formed in ductile mode present as long and thin curled strips with a smooth back surface. With the increasing of cutting length, tool wear aggravates and results in more or less brittle damage in workpieces, which significantly deteriorate the machined surface quality.

## Figures and Tables

**Table 1:** Chemical composition and mechanical properties of the workpiece material.

Chemical Composition (wt %)		Mechanical Properties	
ZrO_2_	<96	Density *ρ* (g/cm^3^)	6.05
Y_2_O_3_	>4	E-modulus *E* (GPa)	210
Al_2_O_3_	<1	Fracture Toughness *K*_IC_ (MPa·m^1/2^)	~10
SiO_2_	<0.02	Vickers Hardness HV10 *H* (kg/mm^2^)	1200

**Table 2:** Tool parameters of the diamond-coated micro end mill.

Tool Parameters	Value
Diameter *d* (mm)	1
Diameter of tool shank *D* (mm)	6
Length of tool *l* (mm)	51
Length of spiral *l*_1_ (mm)	4
Corner radius *r*_ε_ (μm)	8 ± 0.5
Flute number, *z*	2
Helix angle, *θ* (°)	30
Designed rake angle, *α* (°)	2
Designed relief angle, *γ* (°)	14
Cutting-edge radius, *r*_β_ (μm)	7.5 ± 0.5

**Table 3:** The experimental matrix of cutting parameters.

Test No.	Spindle Rotating Speed *n*/rpm	Feed Per Tooth *f*_z_/mm	Milling Depth *a*_p_/mm	Maximum Uncut Chip Thickness *h*_max_/μm
TC1/TP1	38,000	0.001	0.002	0.62
TC4/TP4		0.003	0.002	1.58
TC3/TP3		0.005	0.002	1.99
TC6/TP6		0.001	0.004	0.84
TC5/TP5		0.003	0.004	2.39
TC2/TP2		0.005	0.004	3.55

**Table 4:** Grooves machined at different cutting length for each test sample.

Groove No.	Milling Layers	Cutting Length*l*_c_ (mm)	Cumulative Cutting Length*l*_ac_ (mm)
n1	6	66	66
n2	6	66	132
n3	48	528	660
n4	40	440	1100
n5	40	440	1540
n6	40	440	1980

## References

[1] Ferraris E., Vleugels J., Guo Y., Bourell D., Kruth J.P., Lauwers B. (2016). Shaping of engineering ceramics by electro, chemical and physical processes. CIRP Ann..

[2] Barry C.C., Grant N.M. (2007). Ceramic Materials/Science and Engineering.

[3] Tonshoff H.K., Schmieden W.V., Inasaki I., Konig W., Spur G. (1990). Abrasive machining of silicon. Ann. CIRP.

[4] Uddin A.S., Seah K.H.W., Rahman M., Li X.P., Liu K. (2007). Performance of single crystal diamond tools in ductile mode cutting of silicon. J. Mater. Process. Technol..

[5] Yanyan Y., Bo Z., Junli L. (2009). Ultraprecision surface finishing of nano-ZrO_2_ ceramics using two-dimensional ultrasonic assisted grinding. Int. J. Adv. Manuf. Technol..

[6] Zhao B. (2005). Study on ultrasonic vibration grinding character of nano ZrO_2_ ceramics. Key Eng. Mater..

[7] Liu K., Li X.P., Rahman M. (2003). Characteristics of high speed micro-cutting of tungsten carbide. J. Mater. Process. Technol..

[8] Liu K., Li X., Rahman M., Neo K., Liu X. (2007). A study of the effect of tool cutting edge radius on ductile cutting of silicon wafers. Int. J. Adv. Manuf. Technol..

[9] Fang F.Z., Zhang G.X. (2004). An experimental study of optical glass machining. Int. J. Adv. Manuf. Technol..

[10] Bifano T.G., Dow T.A., Scattergood R.O. (1991). Ductile-regime grinding—A new technology for machining brittle materials. J. Eng. Ind. Trans. ASME.

[11] Yan J., Zhang Z., Kuriyagawa T. (2009). Mechanism for material removal in diamond turning of reaction-bonded silicon carbide. Int. J. Mach. Tools Manuf..

[12] Beltrão P.A., Gee A.E., Corbett J., Whatmore R.W. (1999). Ductile mode machining of commercial PZT ceramics. CIRP Ann. Manuf. Technol..

[13] Zhong Z.W. (2003). Ductile or partial ductile mode machining of brittle materials. Int. J. Adv. Manuf. Technol..

[14] Kumar M., Melkote S., Lahoti G. (2011). Laser-assisted microgrinding of ceramics. CIRP Ann..

[15] Kizaki T., Ito Y., Tanabe S., Kim Y., Sugita N., Mitsuishi M. (2016). Laser-assisted machining of zirconia ceramics using a diamond bur. Procedia CIRP.

[16] Ferraris E., Reynaerts D., Lauwers B. (2011). Micro-EDM process investigation and comparison performance of Al_3_O_2_ and ZrO_2_ based ceramic composites. CIRP Ann. Manuf. Technol..

[17] Lauwers B., Kruth J.P., Brans K. (2007). Development of technology and strategies for the machining of ceramic components by sinking and milling EDM. CIRP Ann. Manuf. Technol..

[18] Dhanorker A., Özel T. (2008). Meso/micro scale milling for micro-manufacturing. Int. J. Mechatron. Manuf. Syst..

[19] Ehmann K.F., Devor R.E., Kapoor S.G. (2002). PL-2 micro/meso-scale mechanical manufacturing—Opportunities and challenges. JSME/ASME Int. Conf. Mater. Process..

[20] Kirsch B., Bohley M., Arrabiyeh P., Aurich J. (2017). Application of ultra-small micro grinding and micro milling tools: Possibilities and limitations. Micromachines.

[21] Williams R.E., Huang Y., Melkote S., Kinsey B. (2005). Recent advances in micro/meso-scale manufacturing processes. Manuf. Eng. Div. ASME.

[22] Torres C.D., Heaney P.J., Sumant A.V., Hamilton M.A., Carpick R.W., Pfefferkorn F.E. (2009). Analyzing the performance of diamond-coated micro end mills. Int. J. Mach. Tools Manuf..

[23] Huo D., Chen W., Teng X., Lin C., Yang K. (2017). Modeling the influence of tool deflection on cutting force and surface generation in micro-milling. Micromachines.

[24] Nabhani F. (2001). Wear mechanisms of ultra-hard cutting tools materials. J. Mater. Process. Technol..

[25] Le Huu L., Schmitt M., Paulmier D., Mamalis A.G., Grabchenko A. (1999). Tribological properties of smooth diamond coatings for cutting tools. Wear.

[26] Zhan Z., He N., Li L., Shrestha R., Liu J., Wang S. (2014). Precision milling of tungsten carbide with micro PCD milling tool. Int. J. Adv. Manuf. Technol..

[27] Takesue S., Katahira K., Komotori J. (2014). A Study on PCD tool surface reconditioning technique for SiC micromachining. Procedia CIRP.

[28] Warhanek M., Pfaff J., Martin P., Schönbächler L., Boos J., Wegener K. (2016). Geometry Optimization of polycrystalline diamond tools for the milling of sintered ZrO_2_. Procedia CIRP.

[29] Matsumura T., Ono T. (2008). Cutting process of glass with inclined ball end mill. J. Mater. Process. Technol..

[30] Bian R., Ferraris E., He N., Reynaerts D. (2014). Process investigation on meso-scale hard milling of ZrO_2_ by diamond coated tools. Precis. Eng..

[31] Cheng X., Nakamoto K., Sugai M., Matsumoto S., Wang Z.G., Yamazaki K. (2008). Development of ultra-precision machining system with unique wire EDM tool fabrication system for micro/nano-machining. CIRP Ann. Manuf. Technol..

[32] Tanaka H., Shimada S., Anthony L. (2007). Requirements for ductile-mode machining based on deformation analysis of mono-crystalline silicon by molecular dynamics simulation. CIRP Ann. Manuf. Technol..

[33] Liu K., Li X., Liang S. (2007). The mechanism of ductile chip formation in cutting of brittle materials. Int. J. Adv. Manuf. Technol..

[34] Liu K., Melkote S.N. (2006). Effect of plastic side flow on surface roughness in micro-turning process. Int. J. Mach. Tools Manuf..

